# Design Pattern Mining Using Distributed Learning Automata and DNA Sequence Alignment

**DOI:** 10.1371/journal.pone.0106313

**Published:** 2014-09-22

**Authors:** Mansour Esmaeilpour, Vahideh Naderifar, Zarina Shukur

**Affiliations:** 1 Department of Computer Engineering, College of Engineering, Hamedan Branch, Islamic Azad University, Hamedan, Iran; 2 Software Technology and Management Research Center, Faculty of Information Science and Technology, Universiti Kebangsaan Malaysia, Bangi, Malaysia; CSIR-Institute of Microbial Technology, India

## Abstract

**Context:**

Over the last decade, design patterns have been used extensively to generate reusable solutions to frequently encountered problems in software engineering and object oriented programming. A design pattern is a repeatable software design solution that provides a template for solving various instances of a general problem.

**Objective:**

This paper describes a new method for pattern mining, isolating design patterns and relationship between them; and a related tool, DLA-DNA for all implemented pattern and all projects used for evaluation. DLA-DNA achieves acceptable precision and recall instead of other evaluated tools based on distributed learning automata (DLA) and deoxyribonucleic acid (DNA) sequences alignment.

**Method:**

The proposed method mines structural design patterns in the object oriented source code and extracts the strong and weak relationships between them, enabling analyzers and programmers to determine the dependency rate of each object, component, and other section of the code for parameter passing and modular programming. The proposed model can detect design patterns better that available other tools those are Pinot, PTIDEJ and DPJF; and the strengths of their relationships.

**Results:**

The result demonstrate that whenever the source code is build standard and non-standard, based on the design patterns, then the result of the proposed method is near to DPJF and better that Pinot and PTIDEJ. The proposed model is tested on the several source codes and is compared with other related models and available tools those the results show the precision and recall of the proposed method, averagely 20% and 9.6% are more than Pinot, 27% and 31% are more than PTIDEJ and 3.3% and 2% are more than DPJF respectively.

**Conclusion:**

The primary idea of the proposed method is organized in two following steps: the first step, elemental design patterns are identified, while at the second step, is composed to recognize actual design patterns.

## Introduction

In software engineering, a design pattern is a general and reusable solution to a frequently occurring software design problem. A design pattern is not an algorithm that can be converted directly into code but rather a template for solving various instances of a general problem. Design patterns are an important tool in software engineering documentation, allowing software developers to reuse previous strategies for resolving recurrent issues. Moreover, it's very important during the re-documentation process, in particular when the documentation is very poor, incomplete or not up to-date [5].

In object-oriented programming, design patterns can increase the reusability of the software libraries and accelerate the development process by providing tested and, proven development paradigms. In addition, mining design pattern instances from system source code or design can greatly help to understand the systems and change them in the future [6].

Design patterns that provide general solutions to various instances of a problem are often repetitive, time-consuming and costly. Each pattern addresses a problem that may occur repeatedly without resolution searching. Design patterns vary in their weights and levels of abstraction [12]. Table 1 provides a classification of design patterns based on their purpose and scope [2].

**Table 1:** Classification of the design patterns.

		Purpose		
		Creational	Structural	Behavioral
Scope	Class	Factory method	Adapter	Interpreter
				template method
	Objects	Abstract factory	Bridge	Chain of responsibility
		Builder	Composite	Command
		Prototype	Decorator	Iterator
		Singleton	Facade	Mediator
			Flyweight	Memento
			Proxy	Observer
				State
				Strategy
				Visitor

The purpose and scope determine the patterns in the various classes of objects. The relationships between the classes and their subclasses are specified using instance level relationships (e.g. aggregation, composition …) and class level relationships (e.g. inheritance). The relationships between the objects are dynamic and can be changed at run time [14]. The purpose of each pattern can be creational, structural, or behavioral, as described below.

- Creational design patterns: these patterns are related to the methods used to create the objects.
- Structural design patterns: these patterns define the ways in which objects and classes can be combined into larger structures.
- Behavioral design patterns: these patterns describe the relationships between the objects.

In computer programming, several patterns may be combined or offered as alternatives. For example, the composite, iterator, and visitor constructs are often used in combination, while the prototype pattern is often an alternative to the abstract factory [2].

Some of the advantages of design patterns are as follows:

- Reusable software and design: the software design patterns are more reusable, flexible, and extensible when design patterns are employed.
- Documentation: the use of design patterns in software documentation allows developers to recognize the structure and design of the application programming interface (API) instantly.
- Communication and teaching: design patterns provide a common language for software designers and analysts and improve the communication between them.

Each design pattern can be divided into several sections based on its template [13]. The template provides a common structure for the pattern information so that the pattern can be understood, used and compared more effectively. Each design pattern consists of a name and classification, intent (also known as the motivation), range of applicability, structure, and list of participants, list of collaborations, list of consequences, implementation, sample code, list of known uses, and list of related patterns.

By using relationships between design patterns can automatically find patterns of different granularity as a pattern that uses many other patterns has a higher granularity then the other [26]. The relationships between patterns allow combining several patterns and helps in understanding the similarities among the relationships [25]. One application of mining relationships between the patterns is related to the principle of modularity that is a specialization of the principle of separation of concerns. Following the principle of modularity implies separating software into components according to functionality and responsibility. In this paper, deoxyribonucleic acid (DNA) sequences are used to mine the patterns, and DLAs are employed to identify the relationships between the patterns. The remainder of the paper is organized as follows.

The first section provides a review of related work on design patterns, and the second section introduces DNA sequences and DLAs. The proposed model is presented in the third section, and the fourth section describes the results of several experimental tests of the model. The paper is concluded in the fifth section.

## Related Work

The Alexander design pattern was introduced into software development in the 1970s [1], and developers soon understood that this pattern could be successfully incorporated into software architecture. Faced with the problem of designing a high-quality building architecture, Alexander introduced the idea of pattern reusability for the first time. He discovered that exemplary buildings have similar design characteristics, known as patterns.

Several studies on design patterns emerged in the 1990s, including a book on the subject [2]. This book introduced the idea of using patterns in software design and presented a standard template for pattern documentation. Boetcher [7] categorized design patterns into creation-, structure, and behavior-oriented patterns based on ideas borrowed from learning theory. Antoniol [8] presented a technique for the detection of structural patterns in design pattern retrieval tools designed for program comprehension and maintenance. Nikolas [9] introduced a design pattern detection method based on similarity scoring. This method detects the similarity between two vertexes rather than between two graphs. To solve this problem, Jing Dong [10] introduced a method known as template matching, which calculates the similarity between sub-graphs. Other researchers introduced design pattern detection methods based on machine learning (see [3], [4], [21], [22] for further information); however, most of these methods are complex in their implementation and computationally expensive [16].

There are several tools to discover design pattern from source codes but there is no point of discovering design patterns from scratch. Current existing tools on pattern mining usually transform the source code into some intermediate representations to reduce the search complexity [6]. Keller [27] uses the SPOOL tool that retrieves design patterns from C++ that recovered Template method, factory method and bridge. Pat system recovered Adapter, bridge, proxy, composite, decorator that it precision is from 14% to 50% which used by Karamer [28]. Another tool that used by Dong [29] was DP-Miner that are based on the use of matrix and weight and recovered Adapter/command, bridge, composite, strategy/state. The precision values reported only for the pattern in JHotdraw range from 91% to 100%. Olsson [30] Reclassified GoF patterns [2] based on the PINOT tool that the case study was on ANT, AWT, JHotDraw, and Swing. Balanyi [32] XML–based language, the Design Pattern Markup Language (DPML), which provides an easy way for the users to modify pattern descriptions to suit their needs, or even to define their own patterns or just classes in certain relations they wish to find but its precision was less than 60%. Lucia present an approach to recover structural design patterns from OO source code, which is based on the use of visual language grammars and parsing techniques and used the DPRE tool for JHotdraw 5.1, JHotdraw 6.0b1, QuickUML, Apache Ant, Swing, and Eclipse JDT (components UI 3.3.2 and CORE 3.3.3) case study. The precision values reported for all patterns from 41% to 87% [31]. Binun [33] present an approach and a tool, named DPJF for implemented pattern detectors. The basis of this method are to routine application of design pattern detection in program comprehension and let DPJF pioneer novel uses of design pattern detection for software quality assessment and improvement. Gueheneuc [34] introduced a tool for design pattern recovery problem and adapt a bit-vector algorithm inspired to bio-informatics. Patterns and software systems to be analyzed are expressed in terms of string representations, which are formed by classes and relationships between them (association, aggregation, composition, instantiation, inheritance and dummy).

De Lucia [35] Eclipse plug-in implementing a reverse engineering tool supporting the detection of design patterns and their implementation variants. The plug-in exploits a technique able to recover design pattern instances by combining static analysis, based on visual language parsing, with dynamic analysis, based on source code instrumentation.

Currently, it is difficult to determine the pattern role and the variant of interaction groups of a design pattern in an UML diagram as the design pattern information is not represented in the interaction diagram. Loo [36], [37] proposed the UML sequence diagram via UML profile to allow designers to define and visualize the pattern roles and the different types of interaction groups for a design pattern.

The relationships between design patterns allow combining design patterns, in order to modularity principles. Unfortunately, it is difficult to identify these relations if they are not explicit in each pattern. In this case, into consideration the growing number of patterns, the manual analysis of design patterns relationships is a daunting activity [38]. Parnas [23] wrote one of the easiest papers discussing the considerations involved in modularization. A more recent work, [24], describes a responsibility-driven methodology for modularization in an object-oriented context [39]. One of the important issues about relationships between design patterns is parameter, function; variable passing that is related to how to apply each design pattern to each others. This principle can be applied to deciding what code goes in a function, when to define a class, or what files to put classes and functions. Several patterns may be combined or offered as alternatives. For example, sometimes creational pattern overlap. There are cases when either prototype or abstract factory would be appropriate. At other times they complement each other: abstract factory might store a set of prototypes from which to clone and return product objects [2]. Abstract factory, builder and prototype can use singleton in their implementations [2]. Abstract Factory classes are often implemented with Factory Methods (creation through inheritance).

### Background on DNA Sequences

Sequence alignment is a method of obtaining the similarity between two sets of data, which can be divided into two sub-methods: double alignment and multiple alignments. Sequence alignment is widely used in bioinformatics for genome sequence difference identification. Any sequence of DNA, ribonucleic acid (RNA), or proteins can be aligned using a variety of bioinformatics algorithms. Sequence mining is one type of data mining, aimed at statistically identifying the pattern in a set of input data. The pattern values are generally assumed to be discrete. In other words, DNA sequence mining is a method of finding the common subsequence in a set of sequences (often two sequences). These ideas are illustrated in the following numerical example [19].

**Figure 1:**
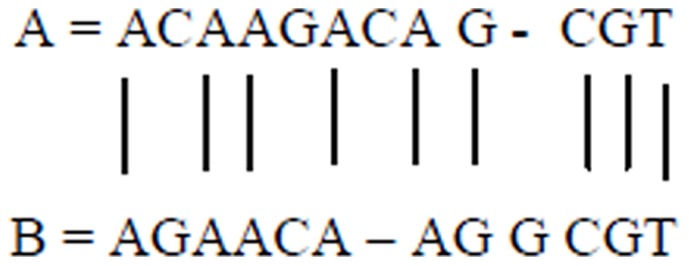
Alignment between sequences *A* and *B*.

Consider the two sequences in Figure 1. The goal is to compare the two sequences and find the similarities between them. The largest common subsequence *S* is defined as the largest sequence such that the letters of *S* appear at the same locations in both *A* and *B* sequences; the letters need not be consecutive in *A* and *B*.

If *A* = *ACAAGACAGCGT* and *B* = *AGAACAAGGCGT*, then Figure 1 shows the alignment (i.e., largest common subsequence) between these two sequences.

The sequence alignment corresponds to the maximum match between the two sequences, assuming that the two sequences are similar.

### Background on Distributed Learning Automata (DLA)

This section introduces the concepts of LAs and DLAs, which are the main tools of the proposed method.

#### Learning Automata

An LA is composed of two parts:

- A stochastic automaton with a specified set of limited actions and a stochastic environment with which the automaton is associated.
- A learning algorithm through which the automaton learns a set of optimal actions by trial and error.

**Figure 2:**
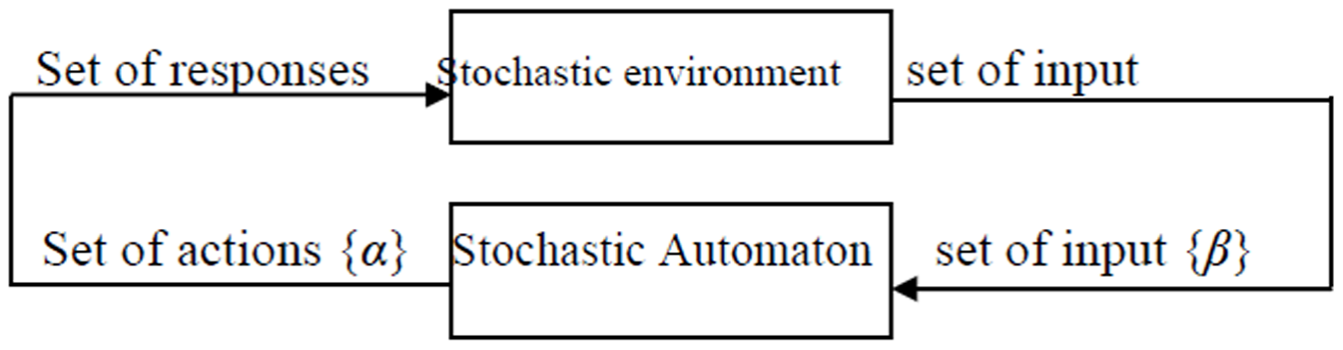
Stochastic learning automaton.

In practice, each action is transmitted to a stochastically generated environment and assessed by that environment; the reaction is then passed to a stochastic automaton [20]. The stochastic automaton uses this reaction to select its action in the next stage [18]. Figure 2 shows the relationship between the stochastic automaton and its environment.

#### The Environment

A formulation of the environment can be derived as follows.

Let *E* = {*α*, *β*, *c*}, where *α* = {*α_1_*,…, *α_m_*} is a set of inputs, *β* = {*β_1_*,…, *β_m_*}is a set of outputs and *c* = {*c_1_*, *c_2_*,…, *c_m_*} is a set of penalty probabilities.

**Figure 3:**
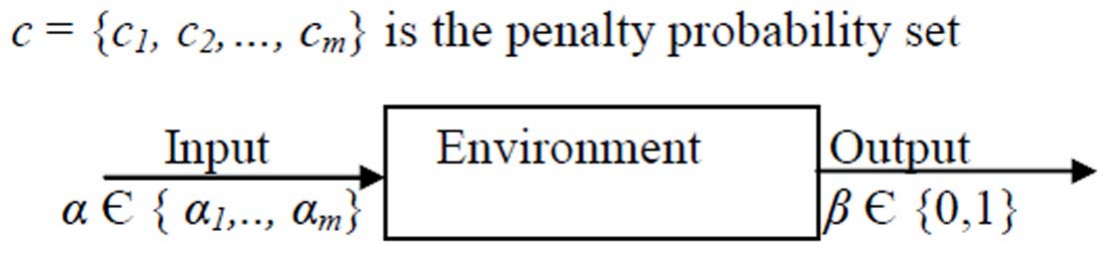
The environment.

Figure 3 illustrates the environment and probability set. Based on the output value (*β*), the formulation of the environment can be divided into the following three cases:

- In the P-Model (probabilistic model), the value of *β_i_* is either zero or one. The value *β_i_*(n) = 1 represents a penalty, and *β_i_*(n) = 0 represents a reward.
- In the Q-Model, *β*_i_(*n*) is selected from a finite set of output with more than two values in the interval [0,1].
- In the S-Model, *β*_i_(*n*) is a continuous random variable in the range [0,1].

The values *c_i_* comprise a set of penalty probabilities and can be used in each of the P-, Q-, and S-models. In a stationary environment, the *c_i_* values remain unchanged, while in a non-stationary environment, these values change over time. The set *c_i_* is defined as follows: 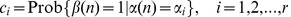
(Equation\;1)


The element *c_i_* represents the probability of an undesirable outcome given the input action *α_i_*. The number of possible actions *α_i_* can vary, but it will be assumed that *c_i_* is defined for at least one action. In this paper, the output value (*β_i_*) of the environment is assumed to be either zero or one; in other words, the P-model learning algorithm is adopted. In this paper, P-model learning algorithm will be used in each node of the distributed learning automata to find the relationship between all existing design patterns in the source codes.

#### P-Model Learning Algorithm

In early models, all of the action probabilities were assumed to be equal for all of the automata. For r-action automata, the action probabilities are given by *p_i_(n)* = *1/r*, where the value of *r* is updated based on the reward or penalty in each iteration. In this type of automaton, if action *α_i_* is selected from among all possible actions, then the desirable outcome occurs, the probability of action *α_i_* increases, and the probabilities of the other actions decrease. However, when an undesirable outcome occurs, the probabilities *P_i_(n)* decrease and the remaining probabilities increase [17]. The changes are made such that the sum of *P_i_(n)* remains equal to one. The following formula defines the effects of desirable and undesirable outcomes.

#### Desirable outcome



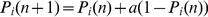
(Equation\;2a)

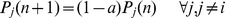
(Equation\;2b)


#### Undesirable outcome



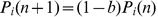
(Equation\;3a)

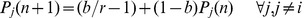
(Equation\;3b)


In Equations 2 and 3, *a* is a reward parameter, *b* is a penalty parameter and *r* is the number of actions such that *a*, *b* 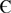 [0,1]. Three distinct cases are considered regarding the values of *a* and *b*.

- L_RP_ (Linear Reward Penalty): when *a* = *b*, the penalty and reward are both important.
- L_ReP_ (Linear Reward Epsilon Penalty): when *b* is substantially less than a (*a>>b*), the reward is more important than the penalty; however, the penalty must still be provided.
- L_RI_ (Linear Reward Inaction): when *b* equals zero (*b = 0*), the penalty is not considered.

#### Distributed Learning Automata

**Figure 4:**
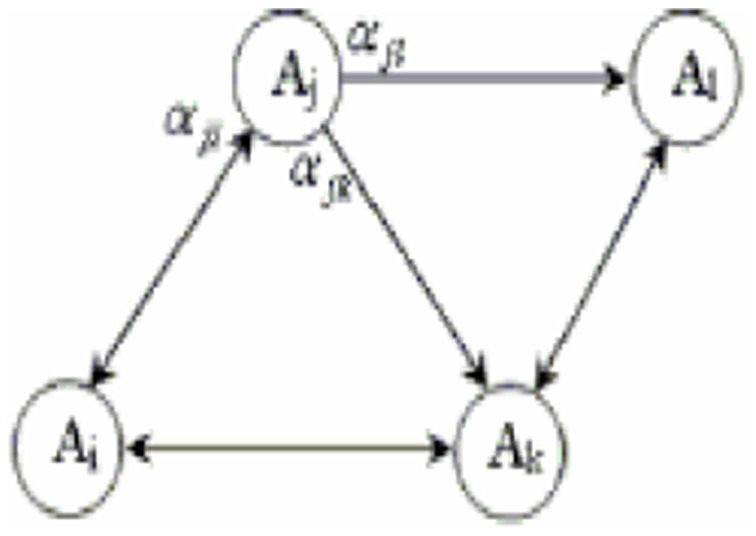
Distributed learning automata.

A DLA is a network of LAs that cooperate with one another in solving problems. In colleague LAs, only one LA is active at any given time. The number of LAs in one DLA is equal to the number of actions that can be performed by any of the other LAs connected to it. The selection of an action by the LAs in this network leads to the isomorphic activation of other LAs connected to this network LA. Figure 4 illustrates the concept of a DLA.

The DLA network is modeled by a graph in which each vertex is an LA. The arrows between LA_i_ and LA_j_ in this graph indicate that the selection of action 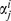 by LA_i_ leads to the activation of LA_j_. The number of actions selected by LA_k_ is determined by 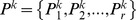. In this set, the value 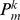 represents the probability of action 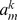, where the selection of action 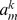 by LA_k_ leads to the activation of LA_m-k_. The values of 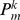 (for all *m*) determine the number of actions that can be performed by the automaton LA_k_ [15].

In this paper, the P-model learning algorithm is used for each LA in the DLA. In this research distributed learning automata is employed to determine the relationships between the patterns in the found design pattern from source codes. The relationships between patterns help in understanding the similarities among the relationships between design patterns.

## The Proposed Method

**Figure 5:**
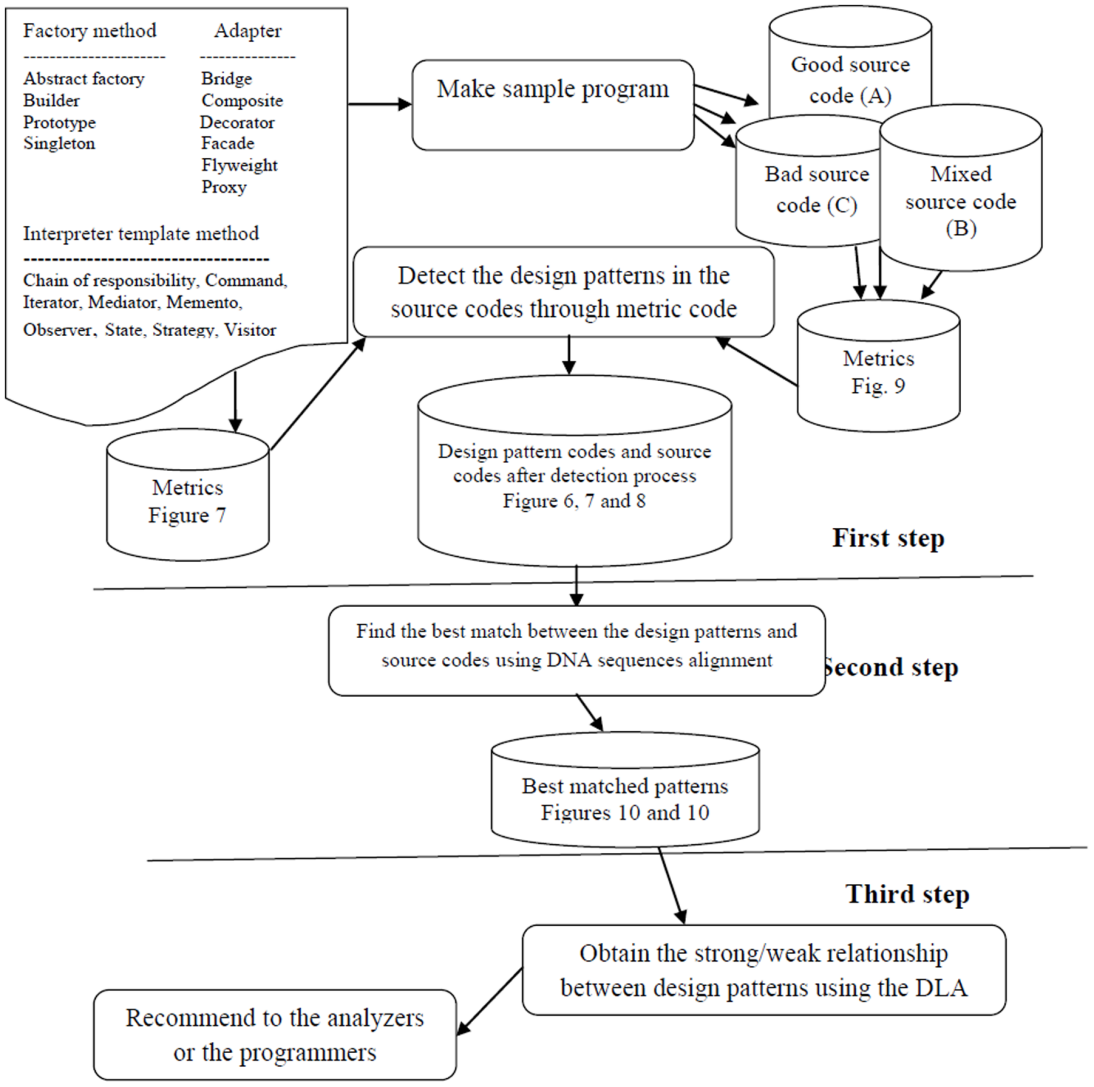
Schematic diagram of the proposed model.

In this paper, DNA sequences are used to detect the patterns, and then, DLAs are employed to determine the dependency rate of these patterns in a specified source code. Figure 5 shows a schematic diagram of the proposed model. According to Figure 5, three categories of source codes are required for the design pattern detection in the proposed model. Three category initial source codes (good, bad, and mixed) are therefore generated using known design patterns. The good source code (A) employs standard design patterns using standard relationships between all objects and classes. The bad source code (C) is the opposite extreme, while the mixed source code employs both standard and non-standard design patterns and relationships.

The standard design patterns and all of the source codes are converted to metric codes using standard symbols, this is done as follows.

- The type of the functions, variables and reserve comments will remain fixing.
- Class name and function name concert to class# and function#.
- Function input parameters and variable names will be considered as representative#.
- Entire internal operations of each function become one sentence and convert to the constant#.

**Figure 6:**
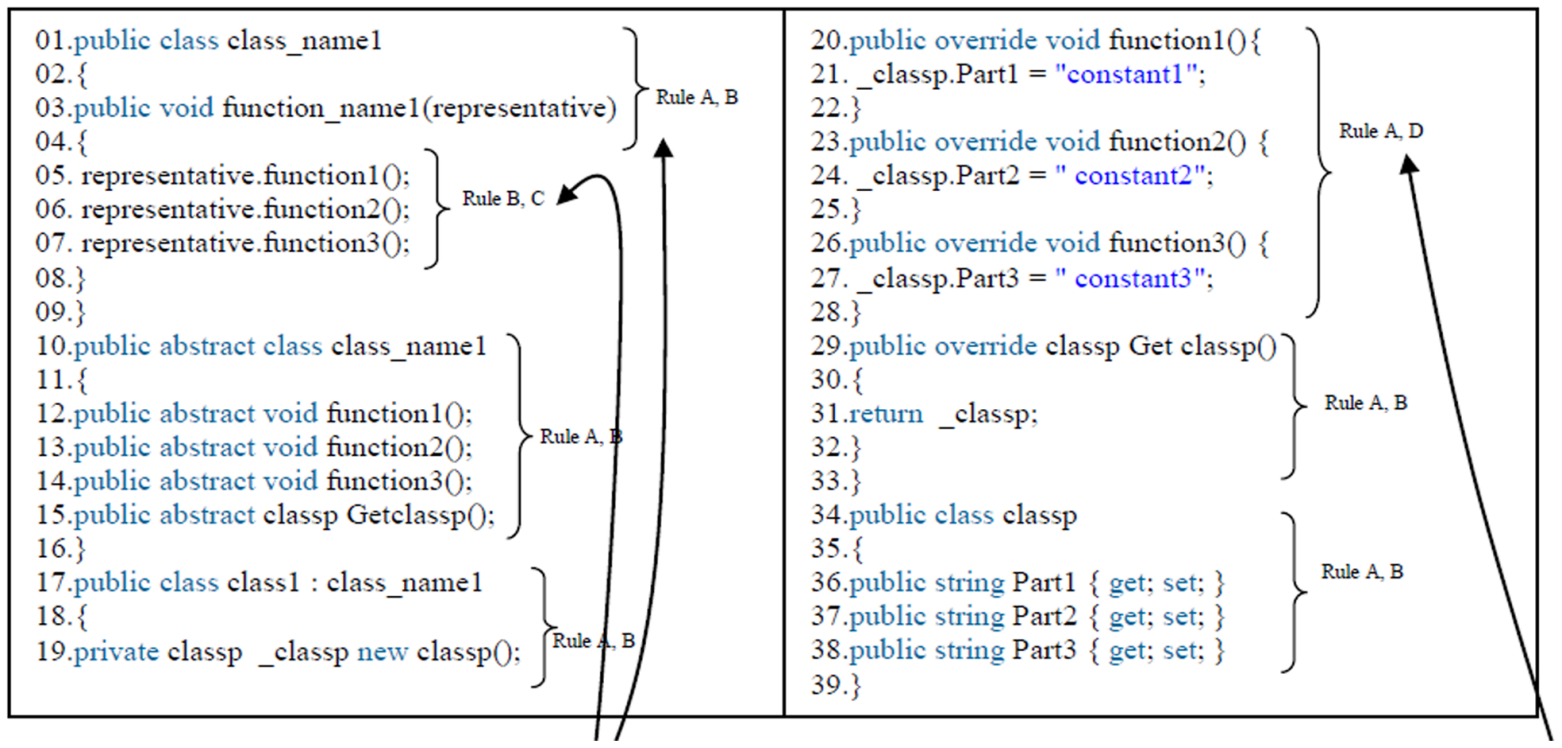
Metric version of the builder design pattern.

**Figure 7:**
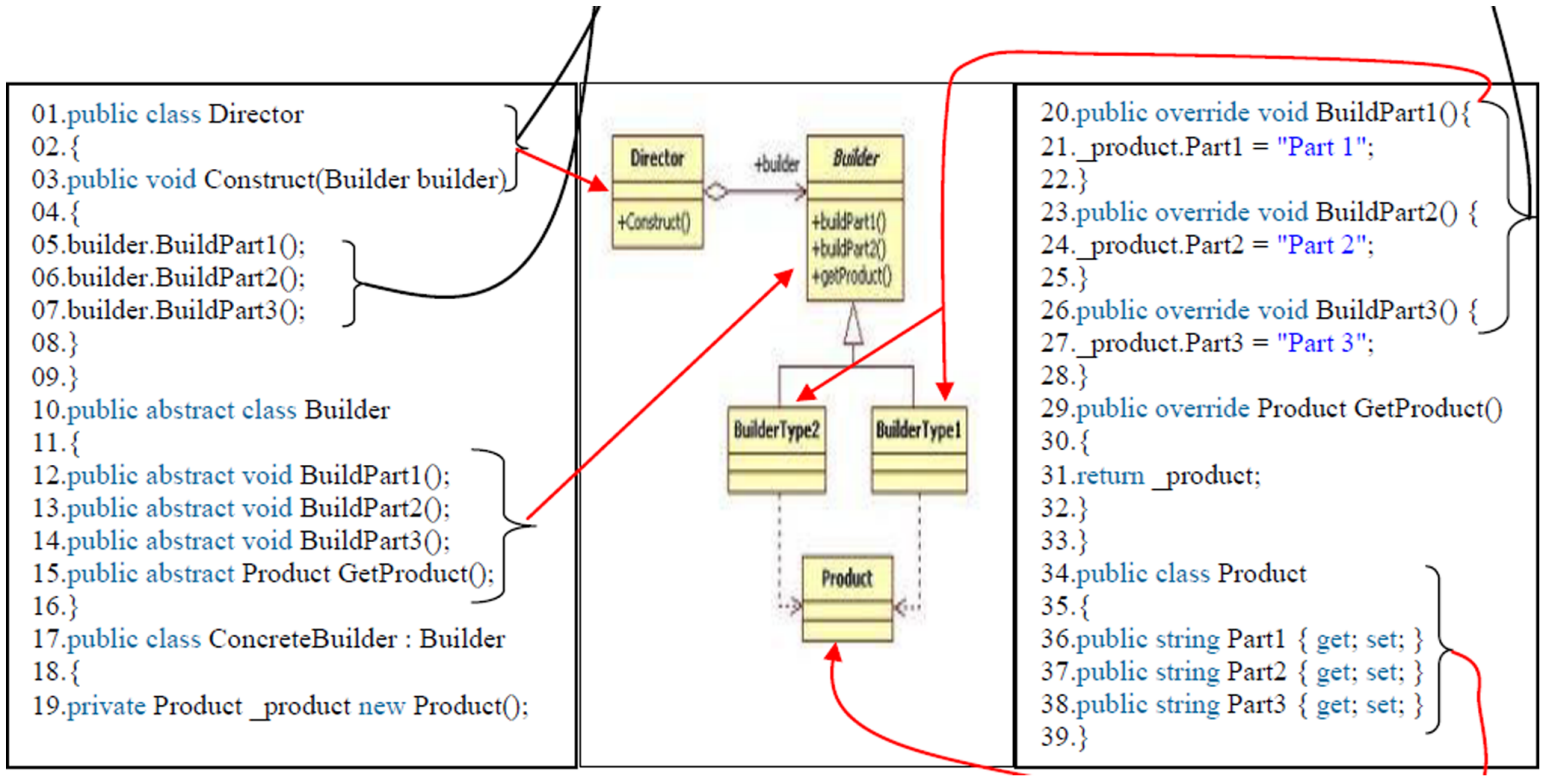
Builder design pattern.

**Figure 8:**
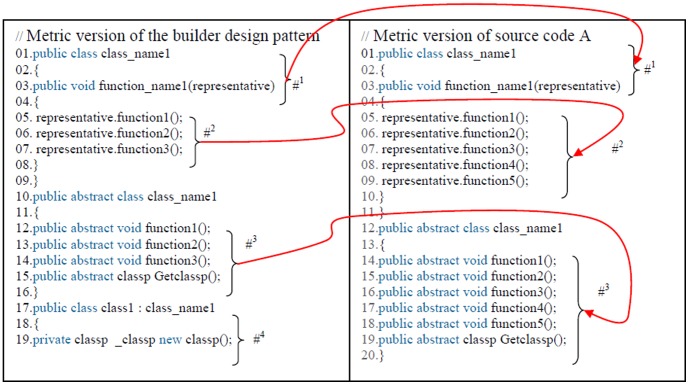
Design pattern and source codes after the detection process.

Figure 6 shows the metric version of the builder design pattern according to previous rules that has been done regarding to the builder design pattern (Figure 7). Following this process, the standard design patterns and all of the source codes are ready for use in the proposed model. Broadly speaking, the proposed model consists of three steps, as described below. Figures 8 shows more example of the source code converting to metric source codes too, regarding to the previous rules.

### First Step: Design Pattern Detection in the Source Codes

Each design pattern has specific properties and characteristics and employs objects and classes with particular variable names and parameters but programmers may change the objects, classes, and names. In this paper, design patterns are therefore characterized based on their structures. Each pattern is converted to metric form so that the pattern design structures in the source codes can be efficiently extracted based on the code variables, parameters, and methods. After the conversion to metric code, the source code is searched for each pattern using the DNA sequence alignment method, which is implemented using dynamic programming [11]. The DNA sequence method is used to identify the largest match between each pattern and a specified section of the source code. If the builder pattern is a design pattern in a source code, then this pattern can be detected using DNA sequence alignment, as described in the second step.

Figure 8 shows that the builder design pattern has been detected in source code A, indicating that source code A has been written following the builder design pattern. The match between the builder design pattern and source code can be seen from the numbered asterisks in Figures 6 and 7.

### Second Step: Finding the Best Match between the Design Patterns and Source Codes Using DNA Sequences

The output of the first step is a set of node (code group). In the first step, each design pattern was compared with each source code section, yielding sequence alignments of various degrees as output. In the second step, the entire set of sequence alignments is reviewed to find the best match between the design pattern codes and source codes using DNA sequence alignment. If a given section of the source code overlaps significantly with a specified design pattern compared to other patterns, then that source code section is labeled with the matching design pattern. The following algorithm is used to perform the second step.

**Figure 9:**
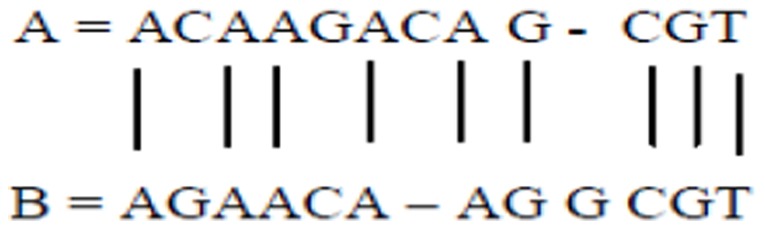
Alignment of sequences *A* and *B*.

- The source code sequence alignment is penalized if space matching occurs. The size of the penalty is one unit. Space matching refers to the scenario in which one sequence element in the design pattern is matched with a space in the source code or vice versa. In Figure 9, sequence *A* exhibits space matching with sequence *B* in nucleotide *G*, and sequence *B* also exhibits space matching with sequence *A* in nucleotide *C*.
- The source code sequence alignment is penalized by three units if non-matching occurs. Non-matching refers to the case in which one element in the first sequence does not match the corresponding element in the second sequence. For example, in Figure 9, sequence *A* exhibits non-matching with sequence *B* in nucleotide pairs *C*, *G* and *G*, *C*.
- After calculating the penalty for each section of the source code, the section with the lowest penalty is selected as a match to the design pattern under consideration. This procedure is performed for all design patterns and source codes. In Figure 9, the size of the penalty for sequences *A* and *B* is equal to eight (3+3+1+1).

**Figure 10:**
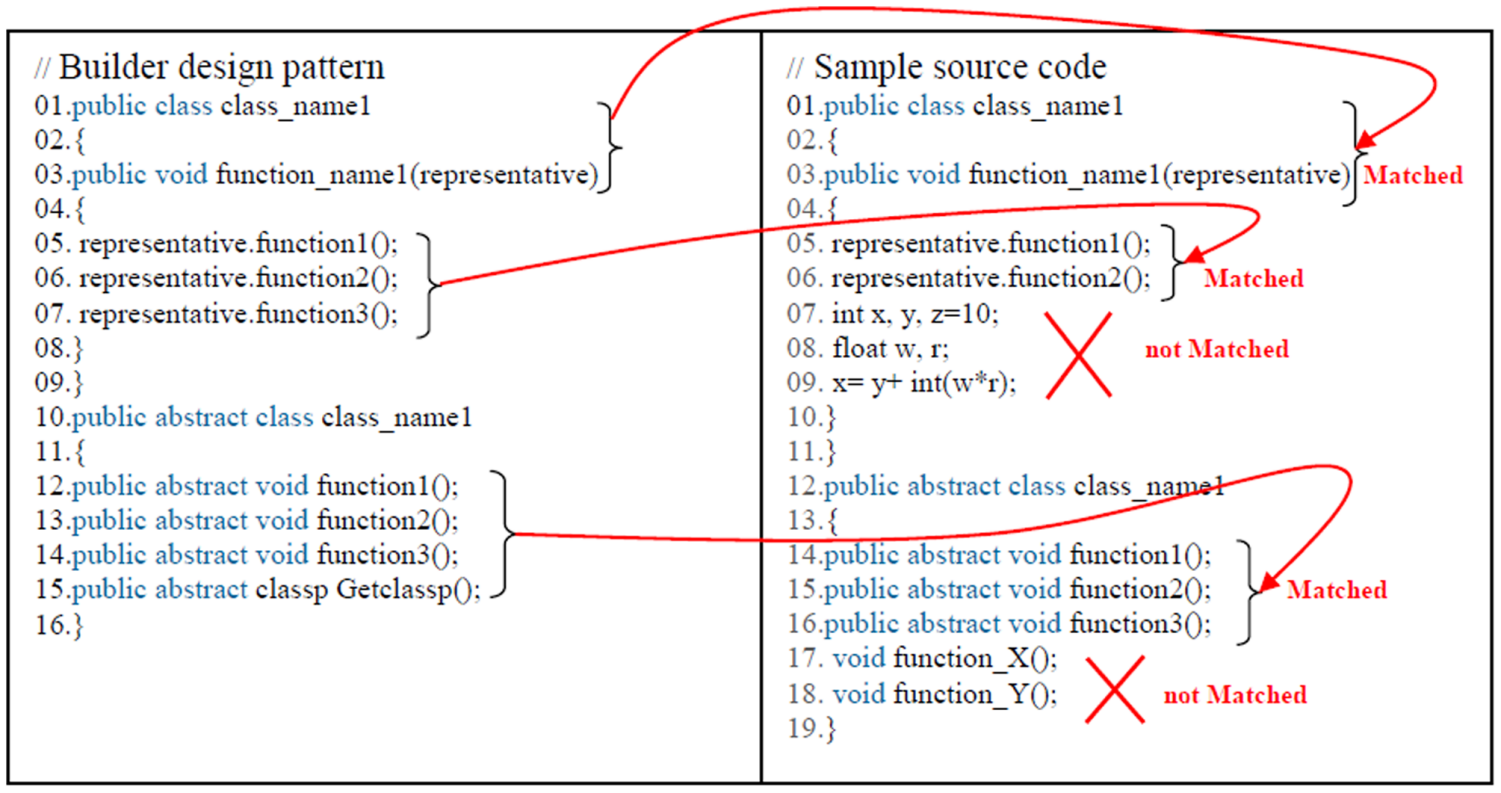
Best matching patterns between the builder design pattern and a sample source code.

Figure 10 shows the sections of the matching pattern between the builder design pattern and a sample source code as bellow.

**Figure 11:**
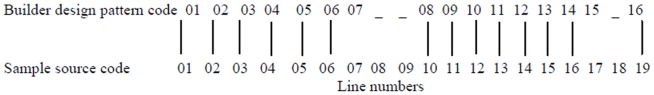
Alignment between the “builder design pattern code” and a “sample source code”.

As well as, Figure 11 shows the alignment between the “builder design pattern code” and a “sample source code” based on Figures 9 and 10.

**Figure 12:**
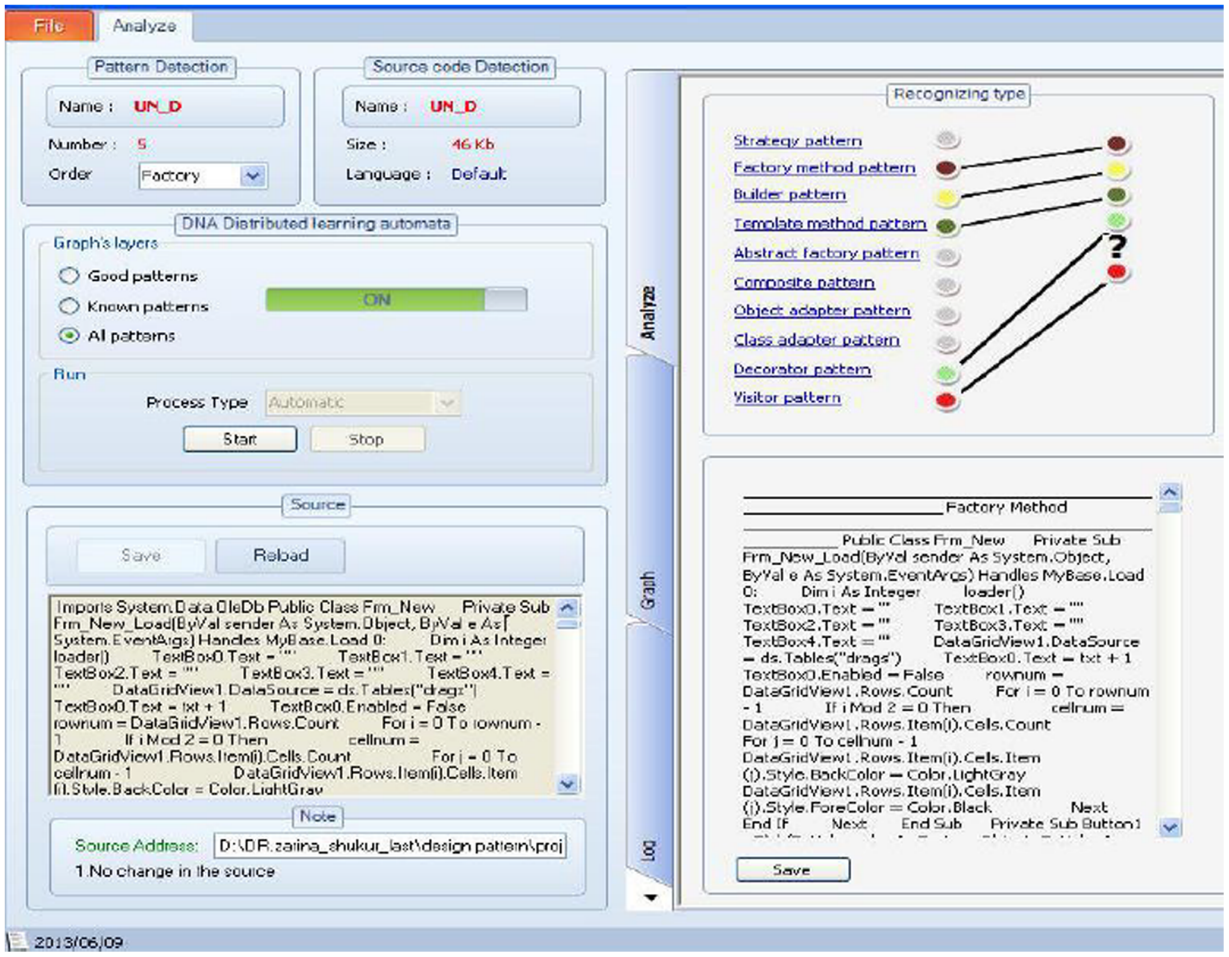
DLA-DNA tool environment for designing pattern detection based on the proposed model.

Design patterns will be detected at the end of the second step as has been shown in the Figure 12. In this case, all sections of a source code will be checked and have been detected standard design patterns. In this paper, DLA-DNA tool is developed according to the proposed method based on the recent three steps. The tool outputs by showing that even better results can be achieved by DNA sequence alignment and distributed learning automata (next step) techniques. The resulting tool is called DLA-DNA (DNA sequence alignment and distributed learning automata).

### Third Step: Extraction of the Weak and Strong Relationship between the Design Patterns

**Figure 13:**
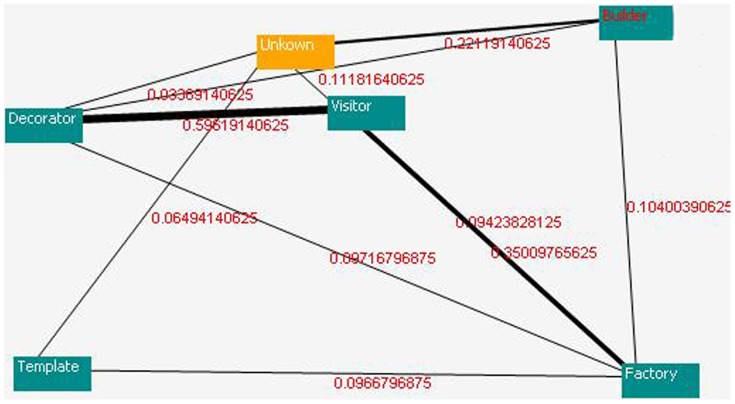
Relationship strengths in source code A.

Following the pattern detection, DLAs are employed to determine the relationships between the patterns. These patterns are investigated based on the send and receive operations, parameters, function calls, classes, and methods. If two patterns have a strong relationship, then this relationship will receive a reward, and the other relationships will obtain penalties. The size of the reward is based on the pair wise similarity between the codes. If the relationship between the two patterns is weak, then both relationships and other relationships will receive a penalty, and the penalties will be assigned to the various relationships based on the pair wise non-similarity of the codes (space matching or non-matching). This procedure is performed on all of the patterns. Figure 13 shows the relationship strengths of the patterns detected in source code A, base on the DLA-DNA tool. The numbers on each connecting line indicate the strengths of the relationships between the pattern pairs in the sample source code.

The relationship between each design patterns in there category of codes will be done as follow. The L_ReP_ case of the P-model learning algorithm is used in the DLA, and *P_i_* is initialized to 1*/n*, where *n* is the number of patterns detected in a source code. For each code pair (see Figure 11), there are three cases. If the two codes are similar, then a reward of *a* = *b*×10 is received. If the two codes are dissimilar because of space matching, then a penalty of *b_1_* = 0.01 is received. If the two codes are dissimilar because of non-matching between two code line numbers, then a penalty of *b_2_* = 0.02 is received. Before calculating the pattern relationships, the net penalty is *b = b_1_+b_2_*, where *a* and *b* are the reward and penalty sizes, respectively. For instance, in Figure 11, there are three space matching instances, yielding *b_1_* = 3×0.01 = 0.03 (for line numbers 08, 09, and 18), and two non-matching instances, yielding *b_2_* = 2×0.02 = 0.04 (line numbers 07→07 and 15→17). The net penalty is therefore given by *b* = 0.03+0.04 = 0.07, and the reward is given by *a* = 0.07×10 = 0.7.

**Figure 14:**
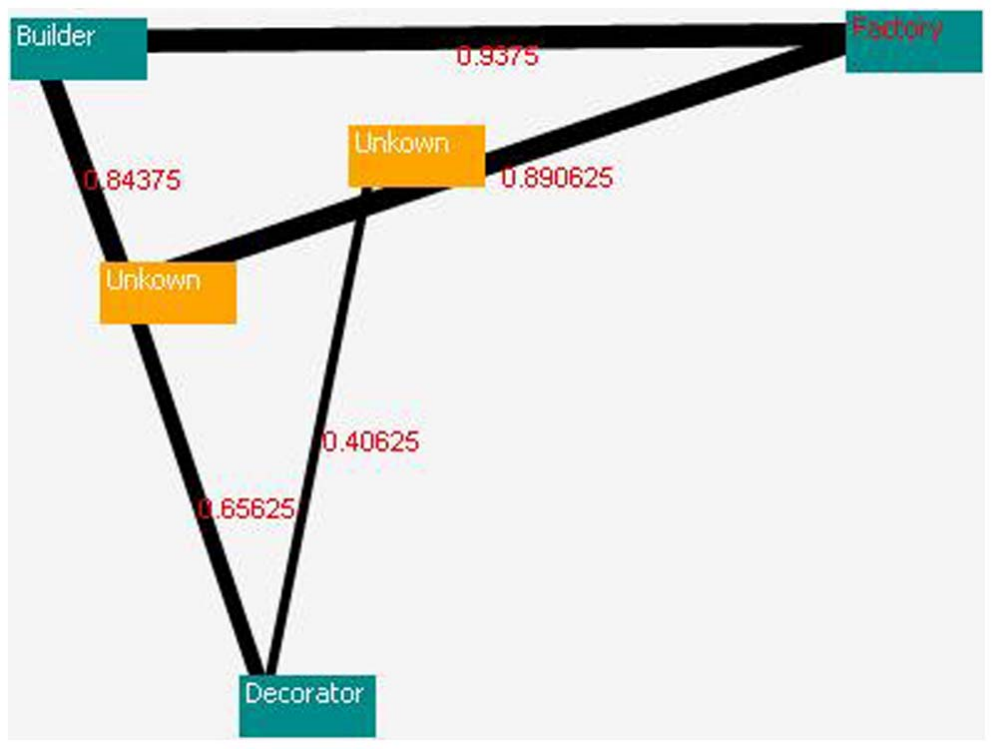
Relationship strengths in source code B.

**Figure 15:**
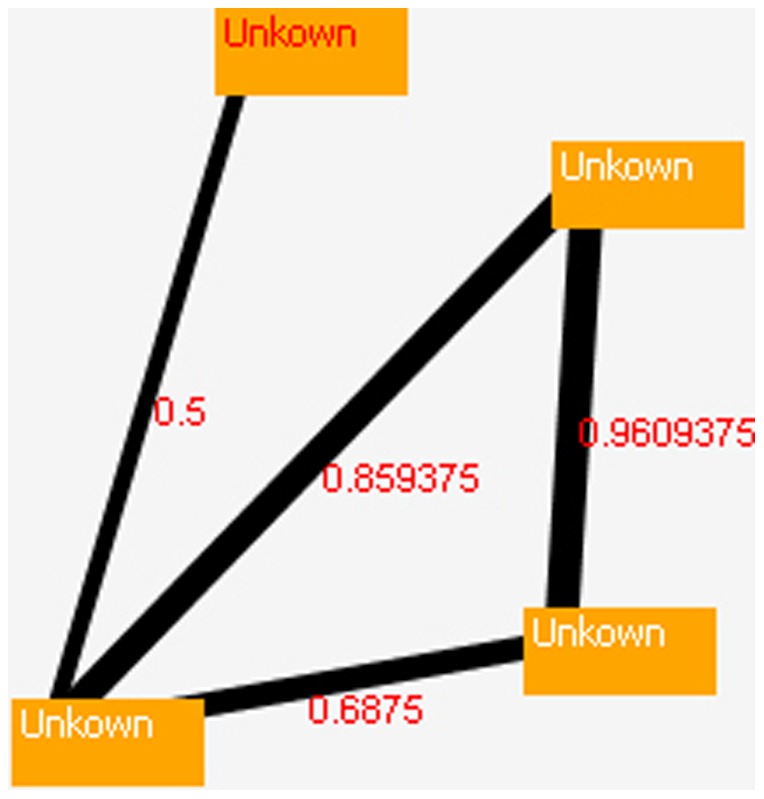
Relationship strengths in source code C.

Figures 13, 14 and 15 show the results of the proposed model for source codes A, B, and C (the way of creation the source codes A, B and C has been expressed in the second paragraph of the experimental result).

Figures 13 to 15 are cut off from DLA-DNA tool environment for more appearance. In Figure 13, the relationship between the detector and visitor patterns is 0.596, and these two patterns have a strong relationship because they have the highest action probability. However, relationship between the template and factory patterns is only 0.097, indicating that the relationship between these patterns is weak, and the code may require modification by an analyzer or programmer. The action probability is different in each source code and depends on the design pattern detection. Figure 15 shows high action probabilities between the various code sections, but in this case, the high values do not indicate strong relationships because the code sections do not follow design patterns, and these high probabilities are therefore ignored. Meaningful strong and weak relationships can only occur between design patterns after their detection. The proposed model can be applied to any source code to detect the standard design patterns in the code and the strengths of the relationships between them. This helps to programmers and analyzers to find out and understanding more about relationship between patterns and its dependence, parameter passing, generalization and so on.

## Experimental Results

In order to assess the precision and recall of the proposed model, the method of 10 fold cross-validation is applied. Five categories of the source codes were used to evaluate the proposed model. These source codes are JHotDraw 6.0b1, Apache Ant 1.6.2, Swing 1.4, ArgoUML [41], and Eclipse 3.6 [42] that the original source codes are in Java. JHotDraw 6.0b1 explain the good use of design patterns and contain many well-known patterns [2], Apache Ant 1.6.2 is a tool for software building process automatically [40], Swing 1.4 is a Sun Microsystems enterprise developer in Java and a set of adjustable graphical components, ArgoUML is a large application and an open source CASE tool. This tool has big size and is a challenge for several tools. Eclipse 3.6 contains very unusual design pattern implementation variants. Table 2 shows some information about these tools from aspect of the number of line of code (LOC), number of packages, number of classes and number of delegations and inheritances.

**Table 2:** Shows some information about five categories of the source codes.

	JHotDraw 6.0b1	Apache Ant 1.6.2	Swing 1.4	ArgoUML	Eclipse 3.6
LOC	19238	79566	125706	154577	5514
Number of packages	171	499	801	200	41
Number of classes	7117	24074	46760	3776	702
Number of delegations and inheritances	7117	24074	46760	20680	2346

In this paper, three category of source code will be used for assessing that is created from a combination of the above source code as bellow.

First category of source code (category A) consists of several standard design patterns with strong relationships of those patterns, the second one (category B) includes both standard and non-standard design patterns of them, and the third source code (category C) consists of several non-standard design patterns. Category A source code is prepared by using the respective five source codes without any modifications, category C is prepared by removing all patterns in the five source codes, and category B code is prepared by combining part of category A source code with part of category C source code. In this experiment, the correctness of the semantic and syntax of the source code are not of our scope and will use the DLA-DNA tool to assess the precision and recall of the proposed model compared with other available tools. Table 3 shows some information about these tools from aspect of the number of line of code (LOC), number of packages, number of classes and number of delegations and inheritances.

**Table 3:** Shows some information about three categories of the source codes.

	Source code A	Source code B	Source code C
LOC	161397	8792	112450
Number of packages	104	8	89
Number of classes	4172	12	155
Number of delegations and inheritances	18627	3	3196

In the first step, all of the design patterns are detected using DNA sequence alignment and fed to the DLA to identify the weak and strong relationships between them (use the DLA-DNA tool). The DLA-DNA tool (proposed method) in comparison to three other tools (Pinot [30], PTIDEJ [34] and DPJF [33]). The results of the precision and recall are as Table 4 and Table 5 respectively. The Similarity of the proposed method and the others is the understand the relationship between patterns; and all of them try to present the notion of elemental design patterns to be employed as base concepts in order to automatically detect more complex design patterns. The primary idea of the proposed method is organized in two following steps: the first step, elemental design patterns are identified, while at the second step, is composed to recognize actual design patterns. But, the difference between the proposed and the others is to describe relationships between objects, methods, and fields for the design patterns to be recognized.

**Table 4:** Precision of the proposed method and other available tools.

Dataset name	Pinot	PTIDEJ	DPJF	DLA-DNA
Source code A	82%	77%	94%	95%
Source code B	61%	52%	86%	93%
Source code C	78%	71%	91%	93%

**Table 5:** Recall of the proposed method and other available tools.

Dataset name	Pinot	PTIDEJ	DPJF	DLA-DNA
Source code A	88%	68%	92%	94%
Source code B	78%	57%	89%	91%
Source code C	82%	59%	90%	92%

Table 4 and Table 5 show the precision and recall comparison on proposed model and some available tools. Regarding to the data selection, three source code those are build based on the standard five source codes. Precision and recall percentages for proposed method and others, is obtained from averaging recovered instances of design patterns (Adapter, Bridge, Composite, Facade, Proxy and Decorator) on source codes A, B and C respectively. Regarding to the Table 4 and Table 5, the precision of the proposed method is better than other related method, especially in the source code B. According to the object oriented programming styles that is much more relevant to characteristics of programmers, they usually does not use the standard design pattern in their programs and usually combine standard design patterns with un-standard programs features. Thus, the proposed method is much better than other from aspect of this feature. The result demonstrate that whenever the source code is build standard and non-standard, based on the design patterns, then the result of the proposed method is near to DPJF and better that Pinot and PTIDEJ. These methods are a latest and newest model on the design pattern detection. Also these methods exploit the code analysis tool source navigator in order to implement the extractor module of the recovery process. Source navigator is able to recover almost all the necessary information and organize it in a structure suitable for our purposes and supports several programming languages, such as C++, Java, and Python [13]; and provides APIs allowing programmers to construct a specific parser.

## Conclusion

The proposed model consists of two main steps. The first step is detection and identification of standard design patterns in the source codes. The second step is determination of the relationships between the detected standard design patterns. The weak and strong relationships between the patterns were extracted using DLA. The proposed model can detect design patterns better that available other tools those are Pinot, PTIDEJ and DPJF; and the strengths of their relationships; this information can be used by analyzers and programmers to evaluate the quality of their programs based on design patterns. The proposed model was run on three categories of source codes, and the results demonstrate the more accuracy of the model in detecting design patterns and determining the strengths of the relationships between them. This method is feasible for any source code because it detects patterns based on structural similarity.

## Discussion

Understanding the relationship between patterns is extensible to improve on several fields such as process mining, website browsing, biochemistry, computational biology, medicine and biological science when it is important to find the relationship between genes, proteins and many other molecules in living organisms [43]. In the future work, author would extend the application of the proposed method on improving the works of Zhou et al. those are related to computational biology [44], [45], [46].
